# High-intensity focused ultrasound (HIFU) versus deep brain stimulation (DBS) for refractory tremor: team DBS

**DOI:** 10.1055/s-0045-1808087

**Published:** 2025-07-06

**Authors:** Flávia de Paiva Santos Rolim, Denise Maria Menezes Cury Portela

**Affiliations:** 1 Universidade de Fortaleza, Faculdade de Medicina, Fortaleza CE, Brazil.; 2 Hospital Geral de Fortaleza, Departamento de Neurologia, Núcleo de Distúrbios do Movimento, Fortaleza CE, Brazil.; 3 Centro Universitário UNINOVAFAPI, Faculdade de Medicina, Teresina PI, Brazil.

**Keywords:** Tremor, Deep Brain Stimulation, High-Intensity Focused Ultrasound Ablation, Movement Disorders

## Abstract

Deep brain stimulation (DBS) has been widely accepted as a powerful tool capable of suppressing tremor by modulating the neuronal circuitry, with long-term adaptability and a profile of low adverse effects. It has been the primary treatment for refractory tremor for decades, with sustained long-term efficacy. Recently, magnetic resonance-guided high-frequency focused ultrasound (HIFU) has emerged as an alternative, prompting comparisons between these approaches. Deep brain stimulation offers long-lasting tremor control in Parkinson's disease (PD) and essential tremor (ET). In addition, it enables us to advance our understanding of brain circuits by integrating neuroimaging, electrophysiology, and connectomics data to map the best stimulation spots. Technologies such as adaptive and directional DBS enable real-time adjustments and greater precision, optimizing results and minimizing adverse effects. Although HIFU shows promising results, it remains an ablative and non-adjustable therapy, contrasting with DBS's dynamic and customizable advances.

## INTRODUCTION

High-frequency brain stimulation has long been recognized as an effective tool for tremor suppression through neuronal circuit modulation. 1 Deep brain stimulation (DBS) therapy garnered recognition for its effectiveness, long-term adaptability, and lower profile of adverse effects when compared to previously-established ablative lesions; it has been an evidence-based mainstream treatment for refractory tremors for decades, with sustained efficacy during long-term follow-up. 2 3 With the recent development of magnetic resonance-guided high-frequency focused ultrasound (HIFU) techniques, a comparison of these therapies is inevitable. 4 5 6

## ESTABLISHED TARGETS AND OUTCOMES IN DBS FOR TREMOR

Tremor is the most prevalent hyperkinetic movement disorder and a central feature of many neurological conditions, such as essential tremor (ET) and Parkinson's disease (PD). It can be very disabling, leading to reduced dexterity, impairment in activities of daily living (ADLs), and social constraints. Advanced therapies, such as ablative lesions and DBS, are options to treat pharmacologically-resistant tremors. The thalamic ventral intermediate nucleus (Vim) is considered the primary target of ET. Unilateral Vim-DBS can lead to an average reduction in tremor between 53.4 and 62.8% after 12 months. In a 5-year follow-up, the reduction in contralateral tremor ranged from 60.3 to 75%, with a significant improvement in ADLs. Bilateral Vim-DBS provides enhanced outcomes in tremor control in different studies, improving from 66 to 78% after 12 months, with sustained efficacy for axial, head, cervical, and voice tremors. In the long term, studies 3 7 8 have demonstrated a 48% reduction in the overall tremor scores in bilateral cases. A systematic review 9 evaluated 45 studies involving 1,202 patients treated with DBS and 477 with HIFU and compared the effectiveness of these approaches in the treatment of ET; the results showed that DBS provided a greater improvement in tremor compared to HIFU, with bilateral DBS being superior to both unilateral HIFU and unilateral DBS. 9 Also highly effective in controlling tremor in PD, DBS primarily targets the subthalamic nucleus (STN) or the Vim. Bilateral STN-DBS provides significant control of tremor, particularly in the off-medication state, with a reduction of up to 80% after 6 months and 1 year. Bilateral Vim-DBS maintained improvements of 73% throughout 1 year and of 69% throughout 10 years, offering long-lasting benefits. 3 10

## ADVERSE EFFECTS PROFILES: IS HIFU SAFER THAN DBS?

The adverse effects of DBS are well established and can be categorized into surgical, hardware-related, and stimulation-related events. Surgical complications include intracranial hemorrhage, which occurs in approximately 2.1% of the cases, and infections, with an average prevalence of 5%. The adverse effects related to brain stimulation are often related to poor electrode positioning and spread to adjacent anatomical structures; however, advances in the design of directional DBS (dDBS) electrodes enable the targeted stimulation of specific neural pathways while minimizing the activation of adjacent structures that could lead to side effects. 11 12

Although HIFU is a non-invasive procedure, it creates irreversible thermally-induced lesions in the brain. It has a profile of adverse effects categorized as procedure-related and lesioning effects on neighboring structures. Transient sensations related to sonication, such as vertigo, headache, and dizziness, are frequent. Ataxia, asthenia, and dysgeusia have also been reported 13 and tend to last up to 3 months, probably due to inaccurate lesion location, compromising the internal capsule. In Vim-HIFU for ET, observational studies 14 indicate a 43% incidence of mild sensory disturbances, and 14% persist after 1 year; gait and balance impairments persisted in 10% of patients after 1 year. In PD, unilateral pallidothalamic tract HIFU revealed approximately 50% of motor improvement in the Movement Disorder Society Unified Parkinson's Disease Rating Scale (MDS-UPDRS), with mild speech disturbances reported in 50% of the cases and drooling, in 20%. 15 Another HIFU target under investigation in PD is the STN, with significant motor control in different series but with more expressive adverse effects. In a 6-month follow-up after unilateral STN-HIFU, dysarthria was reported in 20% of the patients, mild balance and gait issues, in 25%, and dyskinesias, in 25%. Bilateral HIFU lesions in PD may yield better motor outcomes, but they are accompanied by more adverse effects. Some case series 16 17 18 19 have reported persistent speech disorders in 17 to 39% of the cases; postoperative dyskinesias occurred in 72% of the patients, but they were generally transient and controlled within a few months; and ataxia and gait instability were observed in up to 17% of the cases with large bilateral lesions. The main concern of HIFU ablative lesioning within the STN is that, due to its small size, the approach may be unable to cover all the subregions needed to simultaneously treat parkinsonian symptoms. It would be necessary to enlarge the lesion size, which significantly increases the risk of adverse effects due to the impact on adjacent structures or functional connectivity of the STN. Staged procedures are recommended in HIFU treatment to manage potential side effects and evaluate the initial treatment's effectiveness before proceeding with the second side. A standardized protocol has not been universally established, but it is suggested that at least a 6-month interval be maintained. 20

## BALANCING COST-EFFECTIVENESS: DBS versus HIFU

The cost-effectiveness analysis comparing DBS and HIFU for refractory tremor indicates that HIFU is an economically-viable alternative, with initial lower costs (range: $19,000–25,000, versus $62,000–100,000 for DBS) and shorter hospitalization time. Regarding quality-adjusted life years (QALYs), HIFU offers gains similar to those of DBS, with a difference of 0.47 QALY. However, DBS is a technologically-advanced and flexible therapy, as it enables continuous adjustments in stimulation throughout life, also enabling greater symptom control and adaptability to changes in the patient's clinical condition. Although less invasive, HIFU creates a static and irreversible lesion with a recurrence rate of symptoms between 8.3 and 14% throughout 5 years. 21 22

## DBS ENABLES TAILORED AND ADAPTABLE MODULATION OF BRAIN CIRCUITRY

Directional DBS enables coverage of multiple areas, increasing the power of stimulation and consequently ensuring greater effectiveness of the therapy. In ET, patients with severe tremor may require modulation of additional targets, such as the posterior subthalamic area (PSA), or combined stimulation (Vim + PSA), to suppress the tremor. Directional DBS systems seem to provide better tremor control in ET (68% versus 48% in standard DBS), with greater activation of the pallidofugal pathways and more ventral location in “sweet spots,” including the zona incerta (ZI) and posterior subthalamic pathways (PSA). 23 24

Recent studies 24 indicate that connectivity between the contralateral and ipsilateral thalamus strongly influences the stability of tremor, with more unstable tremors being associated with stronger cross-coupling. In the contralateral thalamus, efferent connectivity directed toward the tremor network is predominant, whereas in the ipsilateral thalamus, afferent input originating from the tremor is more pronounced. These findings reveal the different roles of both hemispheres in the generation and modulation of tremors and emphasize the relevance of bilateral integration in the thalamic-tremor network. 24

PD is established as a neurodegenerative disorder in which symptoms progress over time despite optimal clinical or surgical treatments. In addition, growing evidence suggests that ET is a neurodegenerative process that is centered in the cerebellar cortex. Structural changes in Purkinje cells have been identified, 25 with the most affected side of the cerebellum corresponding to the body side with the most severe tremor. These neuropathological aspects strengthen the need for personalized and adjustable therapies to ensure effectiveness over time. A recent blinded and randomized feasibility trial 26 suggested that DBS could alter the natural progression of early-stage PD. Stimulation parameters were adjusted to target the dorsolateral “sweet spot” of the STN, which is functionally connected to the primary motor cortex and the supplementary motor area. This adjustment led to a reduction in MDS-UPDRS Part III scores in the off-medication state. After a 7-day washout period. These findings reinforce the potential of DBS in PD by integrating neuroimaging advances in surgical planning with precise adjustments in programming to optimize clinical benefits over time. 26 Directional DBS makes it possible to precisely shape the delivered electric field to target the most relevant neural circuits, thus minimizing side effects and increasing efficacy. By adjusting the stimulation parameters, it is also possible to simultaneously target different tracts, customize the treatment according to the most bothersome symptoms of each patient, and independently apply different strategies in each cerebral hemisphere. 27 28

## TECHNOLOGICAL BREAKTHROUGHS AND ADAPTIVE DBS: THE FUTURE IS HERE

The technological evolution of electrodes and implanted pulse generators compatible with high-field MRI, combined with the modernization of software for imaging fusion, has enabled us to reshape how electrical stimulation is delivered to patients, with better imaging of targets and volume-activated tissue. DBS has evolved from a surgical technique to an advanced tool to investigate brain circuits. Some of the technological advances include chronic monitoring of local neural field potentials, integrating neuroimaging and connectomics data to map “sweet spots” and optimize stimulation sites, and using closed-loop adaptive DBS with real-time adjustments based on neurophysiological biomarkers. 29 Integrating brain-computer interfaces with DBS enables the interpretation of pathological brain signals, optimizing stimulation in real time. Recent studies explore the use machine learning algorithms to decode brain signals and identify electrophysiological biomarkers that can inform real-time adjustments to stimulation parameters. In addition, applying reinforcement learning techniques to closed-loop DBS systems has shown a potential to optimize energy efficiency and therapeutic effectiveness by reducing energy dissipation and preventing overstimulation. This progress paves the way for increasingly precise, adaptable, and personalized brain modulation therapy. 27 28 30

In conclusion, although HIFU is evolving and presenting encouraging data as an advanced treatment for tremors, it is impossible to consider that, while DBS technology is advancing, HIFU remains an ablative, static, and non-adjustable therapy for progressive neurological disorders—an alternative for patients with contraindications to DBS or those who refuse conventional surgical treatment.
